# Supplementary material for: HCC in the Era of Direct-Acting Antiviral Agents (DAAs): Surgical and Other Curative or Palliative Strategies in the Elderly
Source: Cancers (Basel). 2021 Jun 17;13(12):3025. doi: 10.3390/cancers13123025 (PMC8235445; doi:10.3390/cancers13123025)
Supplement: Supplementary file 1 [file cancers-13-03025-s001.zip › cancers-1221779-SI.pdf]

Table S1. Characteristics of the study population—younger than 65 years.

Variable	HCC-O (*n* = 3)	Without HCC (*n* = 53)	Total (*n* = 56)	P value
Age,y, median [IQR]	54 [52,5-55,5]	54 [51-57]	54 [51-57]	0,913
Male, *n* (%)	3 (100)	39 (73,58)	42 (75)	0,565
Female, *n* (%)	0 (0)	14 (26,42)	14 (25)	
Comorbidities *n* (%)				
Cardiovascular diseases	0 (0)	1 (1,89)	1 (1,79)	1
Kidney failure	0 (0)	2 (3,77)	2 (3,57)	1
Hypertension	0 (0)	3 (5,66)	3 (5,36)	1
Hemoglobin disorders	1 (33,33)	1 (1,89)	2 (3,57)	0,105
ASA				0,0188
I	1 (33,33)	50 (94,34)	51 (91,07)	
II	2 (66,67)	3 (5,66)	5 (8,93)	
III	0 (0)	0 (0)	0 (0)	
HCV Genotype, *n* (%)				1
1	2 (66.67)	32 (60.38)	34 (60.71)	
Non-1	1 (33.33)	21 (39.62)	22 (39.29)	
Child-Turcotte- Pugh score,*n* (%)				1
A	3 (100)	45 (84,91)	48 (85,71)	
B	0 (0)	7 (13,21)	7 (12,50)	
C	0 (0)	1 (1,89)	1 (1,79)	
MELD, median [IQR]	13 [10-15,50]	8 [7-10]	8 [7-11]	0,232
METAVIR, *n*(%)				0,272
F0-1	0 (0)	10 (18,87)	10 (17,86)	
F2	1 (33,33)	8 (15,09)	9 (16,07)	
F3	1 (33,33)	6 (11,32)	7 (12,50)	
F4	1 (33,33)	29 (54,72)	30 (53,57)	
FIB-4, median [IQR]	6,44 [3,71-6,49]	3,34 [1,82-5,1]	3,54 [1,78-5,54]	0,757
Stiffness, median [IQR]	11,70 [9,8-13,35]	14,6 [8,8-17,3]	14,6 [8,58-17,15]	0,649
Steatosis	0 (0)	3 (5,66)	3 (5,36)	1
Platelets, 10^9/l, median [Range]	58 [26-170]	129 [60-300]	126 [26-300]	0,020
AFP pre-DAA, UI/l, median [IQR]	34,80 [18,5-43,05]	1,39 [0,66-3,11]	1,45 [0,69-3,25]	0,033
DAA, *n* (%)				1
SOF Based	3 (100)	45 (84.91)	48 (85.71)	
No SOF Based	0 (0)	8 (10.09)	8 (14.29)	
